# Perforating Fibrous Histiocytoma Mimicking Keratoacanthoma: A Case Report

**DOI:** 10.3390/dermatopathology11010002

**Published:** 2023-12-25

**Authors:** Alina Lungu, Aurélie Hsieh, Gürkan Kaya, Sébastien Menzinger

**Affiliations:** 1 Department of Clinical Pathology, Geneva University Hospitals (HUG), 1205 Geneva, Switzerland; gkaya@hcuge.ch (G.K.); sebastien.menzinger@hcuge.ch (S.M.); 2 Department of Dermatology and Venereology, Geneva University Hospitals (HUG), 1205 Geneva, Switzerland; aurelie.hsieh@hcuge.ch

**Keywords:** perforating fibrous histiocytoma, dermatofibroma, perforating, histopathology

## Abstract

A 31-year-old male presented with a firm, well-demarcated, erythematous, crateriform, and ulcerated nodule in the left lumbar region, which persisted for 3 months. Clinically, a keratoacanthoma was suspected. The histological analysis was consistent with perforating fibrous histiocytoma, a rare histopathologic variant of fibrous histiocytoma. To our knowledge, this is the third case reported in the literature.

## 1. Introduction

Fibrous histiocytoma (FH) or dermatofibroma is a common benign skin lesion, accounting for approximately 3% of skin lesion samples received by dermatopathology laboratories [1]. It usually occurs between the second and forth decades, mostly in females [2] as a slow-growing solitary small plaque or nodule, or sometimes as multiple lesions, and it is associated with a low recurrence rate (2–5%) [3].

Histologically, FH is characterized by a sparsely or highly cellular dermal proliferation of fibrohistiocytic, usually spindle cells, organized in fascicles or in a storiform pattern in a fibrous stroma with a characteristic “entrapment” of collagen fibers at the periphery of the lesion. Follicular or sebaceous induction may be observed in the overlying epidermis, which is usually hyperplastic and hyperpigmented. Depending on the histological variant, the cellularity can vary, and the cells might also be epithelioid, lipidized, atypical, multinucleated, and hemosiderotic deposits, or pseudovascular hemorragic spaces could be observed. Different variants can coexist in the same lesion [4].

In one of the largest retrospective cohort studies published in 2014, which included 192 cases, FH was reported to be most often localized on the limbs (74% of cases). Numerous histopathological variants have been described, the most frequent of which are the following: common FH (80%), aneurysmal (5.7%), hemosiderotic (5.7%), epithelioid (2.6%), cellular (2.1%), lipidized (2.1%), atrophic (1.0 %), and clear cell (0.5%) variants. In this study, no perforating FH was reported [5]. Since they have different probabilities of local recurrence and, in some rare controversial cases, metastasis, the clinical presentation as well as the correct identification of histopathological features of different variants of FH are essential in making the correct diagnosis and in determining the prognosis [5,6]. Multiple FHs, and/or eruptive FH, have been observed in the context of immunosuppression, HIV infection, with highly active antiretroviral therapy (HAART), during pregnancy, in systemic lupus erythematous, and in some acquired perforating dermatosis (APD) as Kyrle’s disease, perforating folliculitis, reactive perforating collagenosis, and elastosis perforans serpiginosa [5,7,8].

The origin of FH remains unclear; nevertheless, there is an increasing number of studies pointing toward a neoplastic origin. Some cases of FH have been found to carry gene fusions involving protein kinase C isoenzymes *PRKCA*, *PRKCB*, and *PRKCD* and occasionally involving the *ALK* gene [9,10]. An overexpression of *FGFR1* (fibroblast growth factor receptor 1), *FGFR2*, and *FGFR4* was also described in some subtypes of FH. Overexpression of FGFRs leads to the activation of MAPK and PI3K/Akt pathways, which is a well-known growth-stimulatory mechanism encountered in benign and malignant tumors [9].

## 2. Case Report

We report the case of a 31-year-old male without any past medical history who presented with a 3-month-standing ulcerated nodule on his lower back, without associated pruritus or history of trauma.

**Figure 1:**
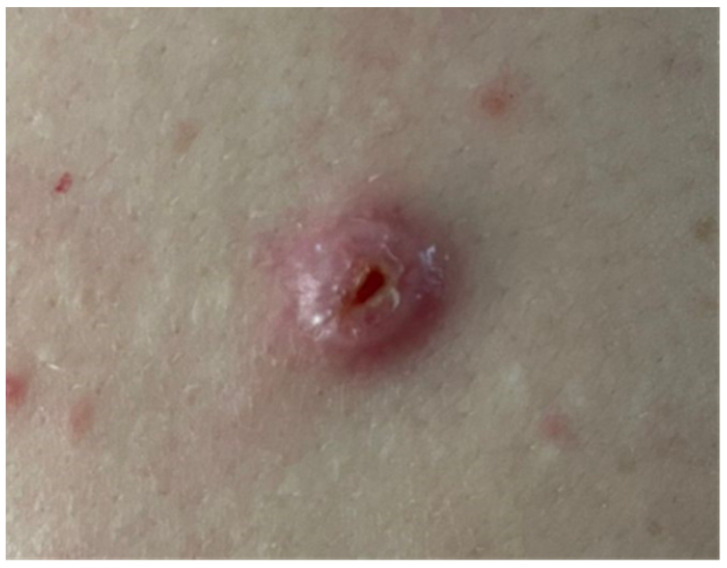
A well-demarcated, erythematous, crateriform nodule with central ulceration.

Clinical examination revealed a firm, well-demarcated, erythematous, crateriform, and focally ulcerated nodule measuring approximatively 1 cm (Figure 1). The clinical differential diagnosis included keratoacanthoma, basal cell carcinoma, and amelanotic malignant melanoma.

**Figure 2:**
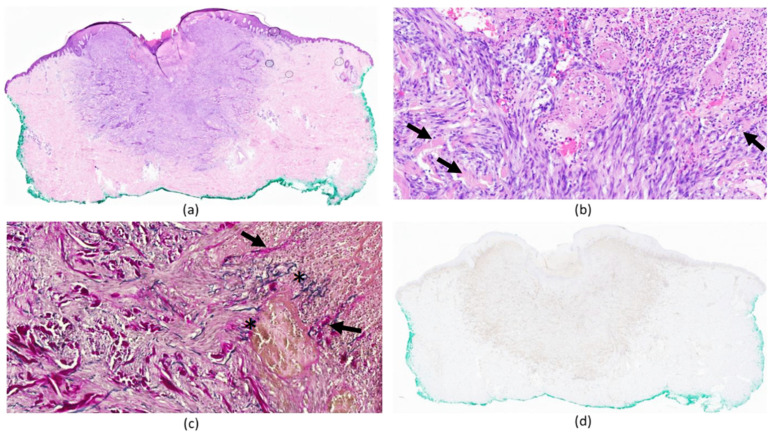
Histopathology of perforating fibrous histiocytoma: relatively well-demarcated cellular infiltrate of spindle cells in the superficial and deep dermis ((**a**) hematoxylin-eosin stain, ×10) with an overlying superficial ulceration, with spindle cells surrounding collagen bundle (pointed out by arrows) that “perforates” the crust ((**b**), hematoxylin-eosin stain, ×30), along with collagen (pointed out by arrows) and elastic fibers (pointed out by asterisks) ((**c**), Miller’s stain, ×30). Factor XIII partial positivity ((**d**), Factor XIII immunostain, ×10).

The histological analysis of the surgical excisional specimen revealed a relatively well-demarcated fusocellular proliferation in the superficial and deep dermis. The lesion was composed of bundles of bland spindle cells arranged in a haphazard to focally storiform pattern (Figure 2a). The spindle cells showed abundant eosinophilic cytoplasm and an oval nucleus with vesicular chromatin without significant cytonuclear atypia. In the periphery, there were entrapped, thickened, globular collagen fibers. The overlying epidermis showed a pseudoepitheliomatous hyperplasia and in the center of the lesion, an ulceration topped with a crust, with some fascicles of spindle cells, collagen, and elastic fibers “perforating” the crust in a perpendicular fashion (Figure 2b,c). There were also rare cells that seemed to perforate the epidermis. The tumor cells were partially positive for Factor XIII (Figure 2d), scanty for CD68, and negative for CD34 and keratins. These histologic features were consistent with perforating FH. The negativity of CD34 ruled out differential diagnoses characterized by a CD34-positive fibrohistiocytic proliferation, including dermatofibrosarcoma protuberans. Moreover, the proliferation index evaluated with Mib-1 (Ki-67) within the lesion was very low (<1%).

## 3. Discussion

We report here a unique case of perforating FH. To our knowledge, only two previous cases have been reported in the literature [11,12]. The first one involved the lower limb of a 12-year-old boy [11], while the second one involved the posterior side of the left auricle of an 82-year-old woman [12].

In the first case, the lesion presented clinically as a 6 mm, firm, nontender, dusky-red to greyish dermal nodule on his left popliteal fossa. It showed a central carteriform aspect, with the exteriorization of dermal components. Histologically, the lesion was located in the upper dermis; it was mostly formed out of epithelioid cells associating with scattered multinucleated giant cells, such as Touton, foreign body, and osteoclast-like types. The lower part contained mainly spindle cells in a storiform pattern with collagen trapping. In the perforating part, macrophages and vertically oriented collagen bundles were described. The presumed explanation for the mechanism precipitating the accumulation of giant cells in FH proposed in this paper was hypoxia and reparative changes in the setting of local irritation [11].

In the second case, the lesion presented clinically as an 8 mm hyperkeratotic erythematous papular lesion on the posterior side of the left auricle with a clinical differential diagnosis including a keratoacanthoma and a squamous cell carcinoma. The excisional biopsy showed a dermal proliferation of fibrohistiocytic cells with a hyperplastic and invaginated epidermis containing keratin and cellular debris, and at the dermal-epidermal junction, it showed fibrin deposits and a sub-epidermal cleavage, suggesting FH accompanied by a perforating dermatosis. No recurrence was detected during her follow-up [12].

We noticed that there were striking clinical similarities between the three cases, leading us to suggest that perforating FH could be regarded as a distinct clinical-pathological entity. This variant should be considered in the differential diagnosis of a crateriform nodule. Table 1 summarizes the main clinical and histological characteristics of each case.

Common clinical differential diagnoses to consider facing a centrally ulcerated, keratotic, plugged, and umbilicated papule or nodule include keratoacanthoma, squamous cell carcinoma, molluscum contagiosum, prurigo nodularis, common wart, etc., but, usually, these entities are easy to distinguish histologically. Nevertheless, some other rare perforating conditions including inflammatory, tumoral, or deposition diseases may present as a single papular, nodular, ombilicated, or crateriform lesion: perforating granuloma annulare [13], involuted Spitz naevus with transepidermal elimination [14], perforating pilomatricoma [15], perforating metastasis from an ovarian adenocarcinoma [16,17], amyloïdosis, calcinosis cutis, osteoma cutis [18], the verrucous variant of perforating collagenoma or ARPC [19], and perforating gout [20].

Several mechanisms are thought to lead to perforation. Trauma from scratching was suggested to be a major trigger of perforating disorders, inducing damage to the epidermis or dermal collagen, resulting in the transepidermal elimination of collagen or elastic fibers. The alteration in collagen or elastic fibers due to metabolic disturbances or microdeposition of various substances represents another hypothesis [7]. Some authors also support the hypothesis that ischemic epidermal alterations may result in the destruction of the epidermis [21].

## 4. Conclusions

Although this is only the third reported case, the striking clinical similarity of the three cases leads us to believe that perforating FH could be a genuine clinical-pathological entity. Perforating FH represents a very rare histologic variant of FH that can present clinically as a crateriform nodule mimicking keratoacanthoma. These are the reasons why we would like to raise awareness of this entity among pathologists, dermatopathologists, and dermatologists. This entity may be possibly under-reported, and further studies are needed to determine whether it also has particular biological and/or molecular characteristics.

## Figures and Tables

**Table 1:** Comparative table with clinical and pathological characteristics of each described case of perforating FH.

Features	Characteristics	Case 1	Case 2	Our Case
Clinical	Gender	Male	Female	Male
	Age (years)	12	82	31
	Site	Back of the knee	Left auricle	Lower back
	Diameter (mm) of the lesion	6	8	10
Histopathological	Epidermal perforation	Present	Present	Present
	Extruded material	Giant cells Macrophages Collagen bundles	Collagen bundles	Collagen bundles Elastic fibers Spindle cells

## Data Availability

The data presented in this study are available on request from the corresponding author. The data are not publicly available due to patient privacy.
